# Role of omics techniques in the toxicity testing of nanoparticles

**DOI:** 10.1186/s12951-017-0320-3

**Published:** 2017-11-21

**Authors:** Eleonore Fröhlich

**Affiliations:** Center for Medical Research, Medical University of Graz, Stiftingtalstr. 24, 8010 Graz, Austria

**Keywords:** Cytotoxicity, Nanoparticles, Omics technologies, Transcriptomics, Proteomics

## Abstract

Nanotechnology is regarded as a key technology of the twenty-first century. Despite the many advantages of nanotechnology it is also known that engineered nanoparticles (NPs) may cause adverse health effects in humans. Reports on toxic effects of NPs relay mainly on conventional (phenotypic) testing but studies of changes in epigenome, transcriptome, proteome, and metabolome induced by NPs have also been performed. NPs most relevant for human exposure in consumer, health and food products are metal, metal oxide and carbon-based NPs. They were also studied quite frequently with omics technologies and an overview of the study results can serve to answer the question if screening for established targets of nanotoxicity (e.g. cell death, proliferation, oxidative stress, and inflammation) is sufficient or if omics techniques are needed to reveal new targets. Regulated pathways identified by omics techniques were confirmed by phenotypic assays performed in the same study and comparison of particle types and cells by the same group indicated a more cell/organ-specific than particle specific regulation pattern. Between different studies moderate overlap of the regulated pathways was observed and cell-specific regulation is less obvious. The lack of standardization in particle exposure, in omics technologies, difficulties to translate mechanistic data to phenotypes and comparison with human in vivo data currently limit the use of these technologies in the prediction of toxic effects by NPs.

## Background

Many scientists view nanotechnology as the revolutionary technology of the twenty-first century because it opened new possibilities for improvement of products used in healthcare, cosmetics, and medicine. Nano-sized materials, on the other hand, can also have negative effects on human health, particularly when inhaled. Epidemiological data showed adverse action of air-borne ultrafine particles on humans, which were confirmed in animal exposures [1]. Toxicity of metal, metal oxide and carbon-based nanoparticles (NPs) is most relevant for human health because exposure to this group of NPs is highest, occurs over long periods and degradation and excretion of the ingested particles are low [2]. Numerous studies have addressed adverse effects of NPs exposure by in vitro and in vivo experiments. The vast majority of in vitro studies used cell-based assays with phenotypic readout parameters, mainly membrane integrity, apoptosis, cell morphology, and proliferation. Oxidative stress was identified as mechanism of toxic action and, therefore, included in the routine testing. Toxicity testing of NPs in vivo comprised exposure of rodents and histopathological evaluation of liver, lung, spleen, kidney, brain, gastrointestinal tract, analysis of bronchoalveolar lavage fluid, blood count and clinical chemistry as readout parameters.

**Fig. 1:**
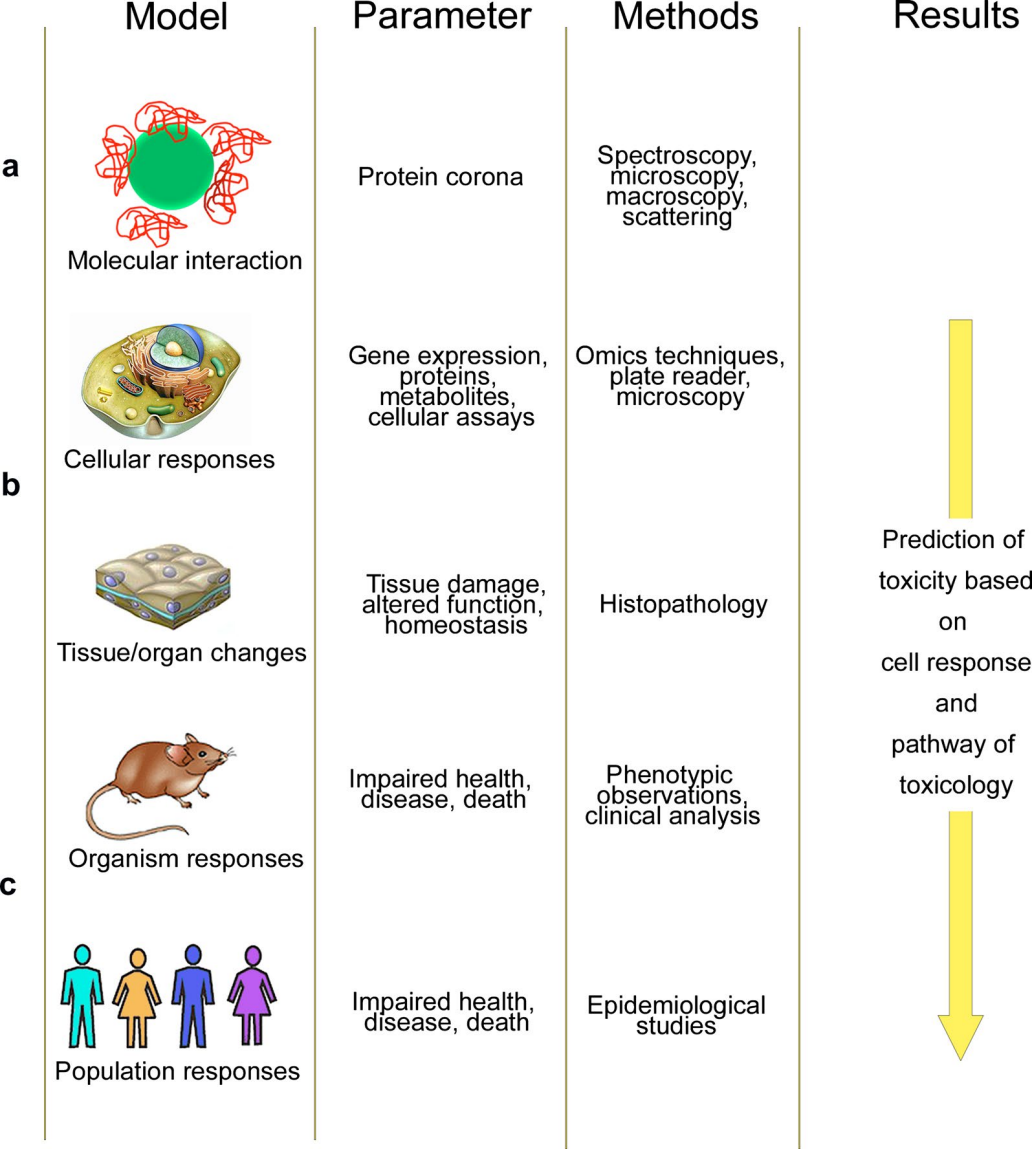
Models, readout parameters and methods in systems toxicology. **a** Analytical techniques to characterize NP—macromolecule interactions include spectroscopical techniques, such as UV–vis spectroscopy, photoluminescence, infrared absorption, Raman scattering, circular dichroism spectroscopy, electron paramagnetic spectroscopy, and fluorescence spectroscopy. **b** Biological assays exploit these technologies and, in addition to that, rely on absorbance, fluorescence and luminescence readers, image analysis and a variety of separation and detection platforms (high-pressure liquid chromatography, gas chromatography, mass spectrometry, nuclear magnetic resonance spectroscopy, electrophoresis, etc.). **c** Further technologies are used for the analysis of organs, mainly histopathology and various staining techniques. Effects on the entire organism can also be detected by imaging techniques (magnetic resonance imaging, ultrasound, computed tomography, radiography, photoacoustic tomography, positron emission tomography, single photon emission computed tomography, thermography) as well as by observation of changes in behavior, appearance, deterioration of health, and death. The predictive value of the obtained results for human toxicology increases from top to bottom

In the last years, principles, methodology and techniques of toxicity testing changed and these developments have also influenced the testing of NPs. One important change was the introduction of quantitative analysis of molecular and functional changes in multiple levels of biological organization in traditional toxicology testing (Fig. 1). The new strategy, termed systems toxicology, changed the current approach of relying almost exclusively on high-dose phenotypic responses in animals [3]. Core technologies in systems toxicology are the “omics” techniques, namely genomics, transcriptomics, proteomics and metabolomics. Omics technologies have also been used for in vitro and in vivo testing of NPs. One advantage might be the identification of new targets and markers for NP toxicity. Such markers would be very useful because exposure to NPs occurs at low levels. If realistic exposure levels are used in conventional in vitro testing it is possible that no phenotypic changes occur because exposure duration is too short. The application of higher doses, on the other hand, may lead to a different cell response because particle agglomeration and stability of the dispersion depend on the particle density [4]. By the use of transcriptomics, however, adverse effects of low particle concentrations on cells may be detected because the techniques identify changes before phenotypic changes are obvious. Another advantage of the omics techniques would be their lower interference with NPs. False positive and negative results in conventional screening assays have been frequently described. They are due to interference by color, fluorescence, chemical activity, light scattering, etc. (e.g. [5]). In contrast, similar problems have not been reported in omics studies. Removal of the NPs during the isolation procedure of the analyte appears to be the most likely reason for that. The use of omics techniques, however, requires more expensive infrastructure and skilled personal in sample preparation and data analysis than conventional testing. Based on the overview of NP studies using omics techniques in vitro and in vivo, this review aims to answer the following questions (i) are omics technologies able to identify new targets in nanotoxicology, (ii) are the technologies robust enough to be used for toxicity screening, (iii) to which extent do the reported regulations correspond to results obtained in phenotypic assays.

NPs contained in commercial products with relevance for human exposure were addressed in this review. They appear suitable for such a comparison although the action of some of the NPs is caused partly by dissolved ions. The different omics technologies are shortly introduced and data within the same study, between platforms and between research groups and NP action across the platforms and phenotypic assays compared. Finally, a comparison of omics technologies to high-throughput phenotypic testing is made.

## Omics techniques

The suffix “omics” stands for “as a whole” and includes epigenomics, genomics, transcriptomics, proteomics and metabolomics. These studies differ from the traditional observation of phenotypes in the way that they can provide primarily mechanistic information and may identify the pathway of toxicity. Based on these techniques it is possible to identify adaptive responses to toxicants at low levels that do not yet cause toxicity but put cells or organisms under stress, which reflects the situation of particle exposure. Identification of cellular stress is important because manifest toxicity occurs when the compensation system is exhausted. An example for the manifestation of adverse effects only upon challenge of the organism is the decreased antibacterial defense of mice that have been exposed to CoO NPs [6].

Toxicants that do not interact or bind to a single type of macromolecule can perturbate multiple pathways and result in a broad activation of pathways. NPs influence various cellular processes (proliferation, apoptosis, inflammation, membrane integrity) [7] and induce such a pattern. In this case, it is usually difficult to deduce the pathway of toxicity from the regulation pattern [3].

Systems toxicology includes genomics, epigenomics (miRNomics and DNA modifications), transcriptomics, proteomics, and metabolomics. Genomics investigates genes and their functions by use of recombinant DNA, DNA sequencing and bioinformatics to analyze function and structure of the genome. The goal is to identify a particular sensitivity of individuals to a given toxin rather than the screening for toxicity of compounds or NPs. The epigenome can be altered by toxicants and, therefore, is useful for toxicity screening.

A detailed description of the respective detection techniques is out of the scope of this review and only the basic principles of the respective techniques will be mentioned.

### Epigenomics–miRNomics

Regulation by miRNAs belongs to the group of epigenetic effects, which are heritable changes in phenotype or gene expression not caused by changes in DNA sequence. MiRNAs are a class of small endogenous non-coding RNAs that, typically, down-regulate gene expression either by interfering with protein synthesis via base pairing (complementary sequences with mRNAs) or by targeting RNA degradation. MiRNAs are produced as primary mRNAs and, still in the nucleus, are processed to pre-mRNAs with stem loop structure. After transfer to the cytoplasm the pre-form matures to RNA duplexes, which release a guide and a passage strand. Only the guide or dominant strand is incorporated in the RNA-induced silencing complex (RISC). The opposite strand (passage or star strand) is quickly degraded. MiRNomics is a relatively new screening platform [8]. MiRNAs are early indicators of cell damage and can be detected in peripheral blood due to slow turnover of the dominant strand. With only around 2000 miRNAs miRNomics might be a better platform for toxicity studies than whole genome expression analysis. The technology is used to identify drug-induced hepatotoxicity, cardiotoxicity, and nephrotoxicity. qPCR profiling of miRNA also identified systemic effects after inhalation of diesel exhaust particles. According to the miRNA profile inhaled PM2.5 induced oxidative stress in asthmatic patients [9]. The physiological relevance of this finding, however, is not yet clear because no correlation of miRNA regulation with airway hyper-responsiveness was seen. The lack of correlation to in vivo findings is one reason why miRNomics is not yet widely used in toxicity screening also of conventional compounds. Toxicologists currently do not completely understand the contribution of miRNA in regulating toxicological outcomes [10].

### Epigenomics—DNA methylation and histone modification

The epigenome further includes DNA methylation, posttranslational modification of histone tails, and chromatin remodeling. DNA methylation is the main mechanism for the down-regulation of gene transcription by preventing the transcription machinery to bind. Its main importance is seen in tumor biology because DNA hypomethylation in tumors is linked to progression and malignancy [11]. Histones are basic proteins that organize eukaryotic DNA into structural units. Binding of histones to DNA is accompanied by decrease of transcription and can be regulated by a variety of post-translational modifications [12]. Histones in the modified state detach from the DNA and, thereby, activate transcription. Increased activity of enzymes that remove these modifications, mainly histone deacetylases, are involved in cancer progression [13]. Epigenetic changes are involved in the transformation and mutation of cells and, therefore, may serve as indicator for genotoxicity. The biological relevance of an altered epigenome is not yet clear because DNA hypomethylation may cause cancer but may also be a consequence of the transformed state induced by altered cell signaling pathways [14]. Therefore, epigenetics is also not (yet) a part of routine pre-clinical evaluation of drugs. Epigenomic studies use a variety of technologies. Histone acetylation is determined mainly based on antibody binding using immunohistochemistry and Western blot. DNA methylation can be quantified by polymerase chain reaction, pyrosequencing, high performance liquid chromatography (HPLC), enzyme-linked immunosorbant assay (ELISA), etc. [15].

Pathways that may indicate adverse effects on DNA are regulation of DNA damage and repair and of nucleic acid metabolism (listed in Table 1).

**Table 1:** Characterization of cell toxicity according to changes in mRNA expression (transcriptomics), proteome (proteomics), and metabolome (metabolomics) with categories

Particle	Size (nm)	Cell	Exp	Regulated pathway(s)	St	Im	De	Pr	Mo	Me	Ve	Si	O	References
A. Transcriptomics														
Ag	5	L51784	3–6 µg/ml; 4 h	Ox. stress, DNA repair	X								X	[76]
Ag	5, 100	U937	1–25 µg/ml; 24 h	5 nm: ox. stress, inflammation	X	X								[80]
Ag	20	Human dermal fetal fibroblasts	3–6 µg/ml; 4 h	Cytoskeleton, energy metabolism, DNA damage					X	X			X	[122]
Ag	20, 30, 60	Caco-2, MCF-7	5–25 µg/ml; 6–24 h	Proliferation, stress response, ox. stress	X			X						[123]
Ag	20, 50	HepG2	2.5 µg/ml; 4–24 h	20 nm: stress response	X									[124]
Ag	20, 50	Human dermal fibroblasts	200 µM; 1–8 h	Cytoskeleton, insulin, HGF signaling, MAPK signaling, ATP content, apoptosis, cytoskeleton			X		X	X		X	X	[52]
Ag	20, 50	A549	1–3 µg/ml; 24–48 h	Cell cycle, ox. stress	X			X						[65]
Ag	20, 50	HepG2	1–3 µg/ml; 24–48 h	Cell cycle progression (low dose), morphological damage (high dose)				X						[75]
Ag	< 100	HeLa	20 µg/ml; 24–48 h	Metabolic process, cellular process, stress response, apoptosis, cell cycle	X		X	X					X	[125]
Ag	100	Embryonic rat cells	20 µg/ml; 48 h	Energy, metabolism, O_2_ transport, inflammation, molecular binding		X				X			X	[69]
Al_2_O_3_	< 100	A549	100 µg/ml; 0–72 h	Cell death, cell cycle arrest			X	X						[81]
Au (NH_2_, COOH, OH)	17–22	Human mesenchymal stem cells	50 µg/ml; 4 h	TFG-β, FGF-2									X	[126]
Au	5, 30	Caco-2	200–300 µM; 24–72 h	Ox. stress, apoptosis, growth inhibition	X		X	X						[127]
Au	20, 34, 61, 113	Caco-2/M-cells	0.5–64 µg/ml; 10–21 days	Ox. stress, ER stress, apoptosis	X		X							[128]

Particle	Size (nm)	Cell	Tech	Regulated pathway(s)	St	Im	De	Pr	Mo	Me	Ve	Si	O	References
A. Transcriptomics														
CdSe/ZnS	8–10	HSF-42	8–80 nM; 48 h	Carbohydrate binding, intracellular vesicle formation, stress response, mitosis, cytokinesis	X			X			X		X	[66]
CeO_2_	3	Caco-2	170 µg/ml; 24–72 h	Mitochondrial function						X				[129]
CuO	50	A549	25 µg/ml; 24 h	Mitosis, cell death, p38 pathway			X	X				X		[130]
CuO (rods, spherical)	12, 50–80	Caco-2	5–100 µg/ml; 24–120 h	Inflammation, ox. stress (rod > spherical)	X	X								[111]
Fe_3_O_4_ (COOH, NH_2_, bare)	14–18	HCM, BE-2-C, 293T	20–80 µg/ml; 2 days	Ox. stress, proliferation	X			X						[131]
Fe_3_O_4_	32	RAW264.7, Hepa1–6	30–100 µg/ml; 4–48 h	Immune effects, cell death, homeostatic processes		X	X			X				[132]
Fe_3_O_4_	32	THP-1, HepG2	50–100 µg/ml; 24 h	Various pathways; HepG2: cell growth, mobility, metabolism				X	X	X				[104]
Fe_2_O_3_, SiO_2_, TiO_2_, ZnO	3, 10, 5, 8–10	RKO, Caco-2, HaCaT, SKMel28	10, 5–50, 1–5 µg/cm^2^; 4 h	Ox. stress response all lines, ZnO in addition protein folding	X								X	[99]
Fe_2_O_3_, SiO_2_, ZnO	100	HEK293	100, 12.5 µg/ml; 24 h	Inflammation, stress, cell death	X	X	X							[107]
MWCNT	5–10 × 20–30	Caco-2, diff. THP-1, small airway cells	10–100 µg/ml; 24 h	Apoptosis, inflammation, cell adhesion		X	X		X					[93]
PEG-silane qdots	10–13	IMR-90	8–80 mM; 48 h	Mitosis, spindle formation				X	X					[66]
SiO_2_	10, 500	RAW264.7	5–50, 250–1000 µg/ml; 24 h	Transcription, cell cycle progression, inflammation response, apoptosis, morphogenesis, differentiation		X	X	X					X	[54]
SiO_2_	12	A549	0.1–6 µg/ml; 24–72 h	Ox. stress response; membrane trafficking; inflammatory response	X	X					X			[78]
SiO_2_	12, 5–10, 10–15	FE1 cells	12.5–100 µg/ml; 24 h	Lysosomal genes							X			[74]
SiO_2_	14, 20	A549	0.05–0.6 mg/ml; 2 h	Inflammation, apoptosis, matrix metalloproteinases		X	X						X	[133]
SiO_2_	67	A549, CCD-34Lu, H2347	0.1–1.5 mg/ml; 24–48 h	Inflammation, signal transduction, cell death regulation		X	X					X		[82]
SWCNT	1–2 × 1000–2000	EAhy926	50 µg/ml; 24 h	Inflammation, ox. stress, and apoptosis	X	X	X							[53]
TiO_2_ anatase	7, 20, 200	HaCaT	n/a; 2–24 h	Inflammation response, cell adhesion		X			X					[55]
TiO_2_, ZnO	12, 15	Jurkat, prim macrophages, DCs	1–10 µg/ml; 6–24 h	Ox. stress related all cell lines, cell death, cell growth, immune system; TiO_2_ inert	X	X	X	X						[94]
TiO_2_	7000 × 200 × 10	Caco-2, diff. THP-1, small airway cells	10–100 µg/ml; 24 h	Apoptosis, cell cycle, inflammation, cell adhesion, phagocytosis		X	X	X	X				X	[93]
WC, WCCo	56, 62	HaCaT	30; 3 µg/ml; 3 h–3 days	Hypoxia-related response, carbohydrate metabolism, endocrine pathways						X			X	[106]
ZnO	20	HaCaT	10–80 µg/ml; 24 h	Apoptosis, ox. stress	X		X							[56]
ZnO	20, 60	A549	25 µg/ml; 24 h	DNA damage, apoptosis, ox. stress	X		X						X	[134]
ZnO	28 × 96, 36 × 95, 44 × 73, 25	Olfactory cells, human stellate cells	2.5–10 µg/ml; 24 h	Stress response, inflammatory response; stress, cell growth and survival, cell signaling	X	X	X	X				X		[135, 136]
A. MiRNomics														
Ag	20	Human neural stem cells	10–200 µg/ml; 6–24 h	Cell cycle arrest, apoptosis, ox. stress, dysfunctional neurogenesis	X		X	X					X	[57]
Ag	30	Jurkat	0.2 µg/ml; 24 h	DNA damage, apoptosis, ox. stress	X		X							[137]
Fe_2_O_3_	3–7	PC-12	214 µg/ml; 24 h	Apoptosis, phagocytosis, inflammation, metabolism, endocytosis		X				X	X		X	[138]
MWCNT	100 × 13,000	BEAS-2B	0.25–2 µg/cm^2^; 1–48 h	Mitochondria, gluconeogenesis, microtubuli function					X	X				[139]
B. Proteomics														
Au	2.2, 5.9, 17	K562 cells	10 mM; 3–24 h	ER stress	X									[79]
Au	5, 15	Balb/3T3	58.8 µg/ml; 72 h	Cell growth, proliferation, morphology, cell cycle, ox. stress, inflammation, ECM synthesis	X	X		X					X	[71]
Ag	20, 200	Caco-2/TC7:HT29-MTX	1 µg/ml; 24 h	Cytoskeleton rearrangement, cell cycle, ox. stress, metabolism	X			X	X	X				[58]
Au	20	Dermal fibroblasts	200 µM; 1–8 h	Signal transduction, cytoskeleton, energy metabolism, ox. stress	X				X	X		X		[140]
Au	20	Small airway cells/MRC-5	1 nM; 72 h	Cell adhesion					X					[70]
NH_2_-Au, CuO, NH_2_-CdTe	20	THP-1	15, 22, 5 µg/ml; 48 h	Topoisomerase (CdTe), ox. stress (CuO), NfkB (Au)	X							X	X	[108]
Au	5, 30	Caco-2	59 µg/ml; 72 h	Metabolism, Energy, transcription, protein, cell morphology and transport, signal transduction, growth, proliferation, antioxidant activity, apoptosis, cell adhesion, cytoskeleton orientation	X		X	X	X	X			X	[59]
Au	20	MRC-5	1 nM; 72 h	Ox. stress, cytoskeleton, cell cycle regulation, DNA repair	X			X	X				X	[141]
Au	20, 100	LoVo	10 µg/ml; 24 h	100 nm: PAK, MAPK, phosphatase 2A pathway; 20 nm: cell stress, protein carbonylation	X							X		[60]
CoO, Fe_3_O_4_, SiO_2_	< 100, 13, 15	RAW264.7	6.25–25 µg/ml; 24 h	ER stress, phagocytosis	X								X	[101]
CuO	30–50	BEAS-2B	0.01 µg/cm^2^; 24–72 h	Maintenance, protein synthesis, death/survival, cell cycle, morphology			X	X		X			X	[72]
Cu, CuO	250	RAW264.7	5–10 µg/ml; 24 h	Ox. stress response, GSH synthesis, cytoskeleton, mitochondrial proteins	X			X		X				[61]
CuO, TiO_2_	22, 25	Murine macrophages	5–10, 100 µg/ml; 24 h	Ox. stress response	X									[77]
Fe_3_O_4_	10, 100	NRK-52E	1 ng/well; 24 h	Cell death related, ras-related, GSH-related, HSP, serpin H1, ER-resident proteins	X		X						X	[142]
MWCNT	(0.6 × 3.6) * 10E3	HEK	0.4 mg/ml; 24–48 h	Metabolism, cell signaling, cell stress, vesicular trafficking, cytoskeleton	X				X		X	X		[143]
MWCNT	(0.1 × 10–20) * 10E3	U937	30 µg/ml; 24 h	Metabolism, biosynthesis, stress response, differentiation	X					X			X	[144]
MWCNT	30 × < 1000	A549	0.3–300 µg/ml, 2–24 h	Proliferation, ox. stress, cytoskeleton	X			X	X					[62]
SiO_2_	25	A549	100 µg/ml; 24 h	Apoptosis, cytoskeleton, ox. stress response, protein synthesis	X		X		X	X				[67]
SiO_2_	15, 30	HaCaT	10 µg/ml; 24 h	Metabolism, ox. stress, cytoskeleton, molecular chaperones, apoptosis	X		X		X	X			X	[68]
SWCNT	1–6 × 1000–2000	HepG2	0.1–100 µg/ml; 24 h	Redox regulation, signaling, cytoskeleton formation, cell growth	X			X	X			X		[145]
SWCNT, (plain, PEGylated)	(0.7–1.6 × 0.2–3) * 10E3	PC-12	0.1–100 µg/ml; 24 h	Antioxidant reactivity, nucleic acid metabolism, lipid metabolism, mitochondrial function	X					X			X	[63]
TiO_2_	18–80	BEAS-2B	10 µg/ml; 24 h	Stress response, metabolism, adhesion, cytoskeleton dynamics, cell growth, cell death, cell signaling	X		X	X	X	X		X	X	[146]
TiO_2_, (coated, plain)	20	Human and rat macrophages	300; 24 h//20 µg/ml; 8–48 h	Metabolic homeostasis, cytoskeleton remodeling, ox. Stress	X				X	X				[73, 147]
TiO_2_	24	A549	2.5–50 µg/ml; 2 month	Glucose metabolism, mitochondrial function, proteasome activity, DNA damage response, p53 activation, cell cycle, proliferation				X		X			X	[148]
ZnO, ZrO	40	J774	10 µg/ml; 24 h	Mitochondrial function, phagocytosis, DNA damage					X				X	[100]
ZnO (Al-doped)	20–40	A549	20–500 µg/ml; 2–48 h	p53 activation, extracellular signaling								X		[149]
C. Metabolomics														
Ag	10, 30, 69	HaCaT	10–100 µg/ml; 24 h	Glycolysis, energy production						X				[150]
Al_2_O_3_	64	Human bronchial epithelial cells	50–500 µg/ml; 24 h	Apoptosis, ox. stress, mitochondrial function	X		X			X				[151]
Au	2	SH-SY5Y	100 ng/ml; 2–6 h	Ox. stress, immune response	X	X								[83]
CuO	28	A549	5–40 µg/ml; 4–24 h	Ox. stress, hypertonic stress, apoptosis	X		X						X	[64]
CuO	< 50	Murine bone marrow MSCs	2–100 µg/ml; 48 h	Serine, glyceric acid, and succinic acid, glutamine						X				[32]
TiO_2_	5	L929	100 µg/ml; 48 h	Amino acid level changes						X				[152]
TiO_2_	< 100	L929	100 µg/ml; 48 h	Carbohydrate metabolism, energy metabolism, mitochondria						X				[153]

Exposure (Exp) with concentration and collection time after treatment with nanoparticles is given. If a range is indicated, several concentrations or time points have been evaluated

### Transcriptomics

The transcriptome represents the entire set of transcripts or mRNAs present in a cell or an organism and is studied by a panel of molecular biological techniques. Gene expression profiling determines the expression level of all mRNAs at a given time point by DNA microarray, next generation RNA sequencing, subtraction hybridization, differential display, or serial analysis of gene expression. Current estimations indicate a number of around 19,000 coded genes [16], which are represented in commercially available whole genome expression arrays. cDNA microarray analysis is the most established omics technique and the testing should ideally be performed across both dose and time. Extracted RNA is subjected to reverse transcription to obtain labeled cDNA or to RNA polymerase amplification to generate labeled cRNA. The sequences are hybridized to oligonucleotides on microarrays and scanned under laser light. After analysis of the hybridization, the identified genes are allocated to pathways based on databases. The advantage of transcriptomics is that only one type of biomolecule has to be extracted and analyzed, compared for instance to proteomics, where different protocols have to be used. A known limitation of transcriptomics is the fact that changes in mRNA expression do not influence the phenotype directly. Transcriptomics is a very established technique with high intra-array reproducibility. Comparison between array platforms, on the other hand, varied with a Pearson correlation coefficient of 0.5–0.95 [17]. Problems include inaccuracy for genes with low expression levels and the fact that not all probes on the arrays match the target genes to the same degree.

### Proteomics

Proteomics describes the analysis of functionally, structurally and anatomically related proteins and can provide more direct information on cellular responses than gene regulation because protective cell responses are often orchestrated through fast modification or changes in cellular localization of proteins. Separation steps to deplete high abundance proteins and chromatographic enrichment help to detect specific proteins and are introduced to improve coverage, sensitivity, reproducibility and throughput of proteome-based analysis. By using two-dimensional electrophoresis around 10,000 distinct proteins can be separated [18]. Fluorescence-labeling or stable isotope-labeling can identify differences in treated versus untreated samples. Analysis can either be bottom-up or top-down. In the first variant peptides released from proteins through proteolysis are analysed [19]. This technique has been termed shotgun proteomics and is widely used. In top-down proteomics intact proteins are analysed. Due to the worse fractionation, ionization and fragmentation in the gas phase, this technique is less universal than the bottom-up technique. Detection uses mainly mass spectrometry (MS) because the platform is relatively flexible and allows the detection of amino acids, peptides and proteins. The mass spectrometer consists of the ionizing source and one or more analyzers. Matrix-assisted laser desorption ionization (MALDI) and electrospray ionization (ESI) are most commonly used for ionization of the molecules, which are then accelerated into time of flight (TOF), ion trap, quadrupole, orbitrap or Fourrier transform ion cyclotron resonance (FTIR) analyzer. Analyzers are usually used in tandem (MS/MS) to achieve higher degree of ion separation and identification. As for transcriptomics calibration and analysis based on proper databases is essential for data interpretation. Due to the detection technique, which includes digestion of the proteins, databases list between 15,000 and 42,000 proteins and between 100 and 2000 millions of peptides [20]. Limitations of proteomics are both biological and technical. The preparation is prone to contamination, and protein expression varies in response to circadian cycles, age, sex and disease. In addition to that, there are many proteins with partly unknown functions; the sensitivity of MS is still lower than other protein detection techniques (e.g. ELISA or Western blot), and usually only water-soluble proteins in a limited range of mass and isoelectric point are analyzed.

In addition to identification of regulated pathways, proteomics plays a specific role for particle characterization because it has been used to characterize proteins that are absorbed to the surface of NPs. The coverage of surfaces with macromolecules, predominantly proteins, is usually referred to as “protein corona” [21]. The binding of the macromolecules affects dispersion of particles in physiological fluids and consists of a relatively stable “hard” layer, which forms within seconds and a less stable “soft” layer that forms within minutes to hours [22]. The composition of the layer depends on absorption and desorption of macromolecules, where the velocity of desorption is the inverse of the velocity of absorption. The two corona layers appear to have different roles for the biological response. The hard layer is resistant and still present after cellular uptake by endosomes, while the soft layer is less stable and determines uptake and biological responses. Various groups studied the protein corona composition using proteomics. Influence of material, particle size and surface charge, hydrophobicity/hydrophilicity, incubation time and type of biological fluid has been reported [23–29]. The studies reported qualitative and quantitative differences in the composition of the protein corona but also a common set of bound proteins. Cytotoxicity of NPs possessing a protein corona was generally lower than toxic effects of NPs without. It is not clear whether a decreased interaction with plasma membrane and decreased production of reactive oxygen species or specific molecules within the layer cause this effect. A link of specific proteins within the protein corona to cytotoxicity has not been identified so far.

### Metabolomics

Compared to the transcriptomics and proteomics, which provide information of potential hazards, metabolomics identifies phenotypic changes that occurred in the presence of the toxicant by measuring changes in carbohydrate, lipid, and amino acid patterns. Metabolomics differs from the former techniques in the way that it is not organism-specific and does not have a fixed code [30]. Metabolomics profiling assesses changes in the entire metabolome and is performed either as footprint (analysis of extracellular metabolites) or as fingerprint (analysis of the intracellular metabolites). To distinguish between these two profiles it is important to prevent leakage of metabolites from cells. Washing may not be ideal because it delays sample processing, which is crucial in order to prevent changes of the metabolite profile after the sampling. The basic workflow including separation and enrichment of the analyte proceeds in a similar way as for proteomics. While analysis by Nuclear Magnetic Resonance (NMR) can detect a variety of metabolites in relatively crude preparation with high reliability, the technique is relatively insensitive and only < 100 metabolites can be detected. MS based techniques are usually preferred because of the higher sensitivity. Separation of the metabolites uses gas chromatography (GC) and liquid chromatography (LC). GC is the ideal method for volatile samples; non-volatile samples can be detected after derivatization. LC can easily separate non-polar metabolites, while polar metabolites may require derivatization. The identity of the metabolites is established by MS–MS fragmentation and comparison of the resulting fragmentation spectra to a reference database. Inter-experiment comparability needs “house-keeping” metabolites or isotope-labeled standards. The relative inexpensiveness of the analysis, the non-invasiveness of the sampling, the low number of metabolites and the good knowledge of the role of most metabolites in the organism make metabolomics particularly suitable for the study of toxicology [31]. There are several limitations to this technology too. The metabolites are not organism-specific and the concentration range can span at least six orders of magnitude. This range cannot be easily compensated because amplification of the signal is not possible. Furthermore, different detection techniques have to be used because metabolites belong to different classes of molecules [30]. Therefore, many studies do not analyze the entire metabolome but use metabolic target analysis or metabolomics profiling, where the analysis is restricted to metabolites of a specific pathway or to a specific group of molecules (for instances lipids). The number of metabolites which is usually detected ranges between 2000 and 7000, although 42,000 metabolites have been entered in the Human Metabolome Databank. Metabolomics identified differences in cellular effects induced by NPs and by microparticles. In the free metabolite screening of human bone marrow mesenchymal stem cells treated with CuO particles the increase in glutamine could discriminate nano-from microparticles [32].

## Omics data in nanotoxicology

Combinations of the keywords “nanoparticles”, “silver, “gold”, “silica”, “titanium dioxide”, “copper oxide”, “zinc oxide”, “carbon nanotubes”, “toxicity”, “nanotoxicity”, “whole genome expression analysis”, “epigenetics”, “proteomics”, “transcriptomics”, “metabolomics”, “miRNA analysis”, and “miRNomics” were used for searches in PubMed and other search machines. For the overview of the in vitro results, particle (material and surface functionalization), particle size, exposure dose and duration, cells used for the studies, and reported regulated pathways are summarized in Table 1. Table 2 contains indication of particle (material and surface functionalization), particle size, animal species, exposure route, exposure dose and duration, and reported regulated pathways reported in animal studies.

**Table 2:** Characterization of in vivo toxicity according to changes in mRNA expression (transcriptomics), proteome (proteomics), and metabolome (metabolomics) with categories

P	Size	Sp	Appl	Exp	Regulated pathway(s)	St	Im	De	Pr	Mo	Me	Ve	Si	O	References
A. Transcriptomics															
Ag	20	Rat	Inhal	381 µg/m^3^; 12 weeks	Kidney: cell cycle, xenobiotic metabolism, extracellular signaling				X					X	[154]
Au	4, 100	Mouse	Iv	426 mg/kg; 30 min	Liver: apoptosis, cell cycle, inflammation, metabolic process		X	X	X		X				[155]
CNT	4 × 67, 0.8 × 11, 3.8 × 49, 5.7 × 49	Mouse	It, oroph, inhal,	Meta-analysis	Lung: inflammation resembling different disease pattern		X								[51]
Cu	25	Rat	Oral	50–200 µg/kg; 5 days	Kidney: coagulation, cell signaling, amino acid metabolism						X			X	[84]
C60, NiO	60, 59	Rat	Inhal	0.12 mg/m^3^; 3 days–4 weeks	Lung: C60: immune process; NiO: ox. stress, inflammation	X	X								[156]
SiO_2_ (Cd-doped)	20	Rat	It	1 mg/animal; 7–30 days	Lung: circadian rhythm, inflammation, cell cycle		X		X					X	[157]
TiO_2_	5–6	Mouse	Ig	10 mg/kg; 90 days	Ovary: estradiol, progesterone metabolism									X	[88]
TiO_2_	5–6	Mouse	Ig	10 mg/kg; 90 days	Liver: inflammation, apoptosis, ox. stress, metabolic process, cell cycle, signal transduction, cytoskeleton, cell differentiation	X	X	X	X	X	X		X		[87]
TiO_2_	5–6	Mouse	Oral	2.5–10 µg/kg; 90 days	Spleen: inflammation, apoptosis, ox. stress, metabolic processes, ion transport, signal transduction, cell proliferation/division, cytoskeleton		X	X		X	X		X	X	[89]
TiO_2_	8, 20, 300	Mouse	It	18–486 µg/animal; 1–90 days	Lung: inflammation, all same pattern		X								[85]
TiO_2_	10, 20.6, 38	Mouse	It	18–486 µg/animal; 1–28 days	Lung: inflammation		X								[158]
TiO_2_	10.5, 10, 20.6	Mouse	It, oroph, inhal,	Meta-analysis	Lung: inflammation resembling different disease pattern		X								[51]
TiO_2_	20.6	Mouse	Inhal	42 mg/m^3^; 1–22 days pn	Liver of offspring: females retinoid pathway									X	[90]
TiO_2_	20.6	Mouse	It	162 µg/animal; 1–22 days	Lung: inflammation		X								[86]
B. Proteomics															
TiO_2_	< 25	Mouse	Ip	100 µg/animal; 7 days	Lung: ox. stress	X									[159]
TiO_2_	< 25	Mouse	Ip	100 µg/animal; 7 days	Liver: inflammation, apoptosis, ox. stress	X	X	X							[160]
TiO_2_	< 25	Mouse	Ip	100 µg/animal; 7 days	Brain: ox. stress	X									[161]
TiO_2_	< 25	Mouse	Ip	100 µg/animal; 7 days	Kidney: ox. stress, signal transduction	X							X		[162]
TiO_2_	25	Mouse	Id	5 µg/animal; 24 h	Lymph node: inflammation, lipid metabolism, mRNA processing, nucleosome assembly		X							X	[163]
ZnO	35	Rat	Inhal	12.1 mg/m^3^; 24 h	Lung: S100A8, S100A9, inflammation		X								[164]
C. Metabolomics															
MnO	10	Rat	Iv	10 mg/kg; 6–48 h	Plasma, urine, tissues: lipid, energy metabolism, amino acid metabolism						X				[165]
PS, lipid polymeric	50, 40, 143, 160, 165	Mouse	It	200, 500 µg/animal; 24 h	BAL: inflammation (all, hydrophobic > less hydrophobic)		X								[91]
ZnO	35, 250	Rat	Inhal	1–5 mg/kg; 24 h	BAL, lung: cell anti-oxidation, energy metabolism, DNA damage and membrane stability	X					X			X	[166]

Application (Appl) and Exposure (Exp) with dose and duration of treatment with nanoparticles is given. If a range is indicated, several concentrations or time points have been evaluated

In the vast majority of studies NPs had an effect on regulation of genes, proteins or metabolites and only few studies reported no effects of NPs. For instance, gold NPs had no significant effect on gene regulation of human vein endothelial cells [33] and no alterations of the protein expression profile was observed in adipose tissue derived stem cells after exposure to 900 nm superparamagnetic iron oxide particles [34].

As the reporting of regulated pathways/processes is not standardized, in order to compare regulated pathways between omics techniques and phenotypic assays the following groups have been formed. *Stress*: oxidative stress, stress response, antioxidant reactivity, GSH-related, HSP, ER stress, cell stress, chaperones; *immune*: inflammation, immune response, phagocytosis; *death*: apoptosis, cell death; *proliferation*: mitosis, growth, proliferation, differentiation, transcription; *morphology*: cytoskeleton, adhesion, mobility, cytoskeleton organization; *metabolism*: mitochondria, ATP content, homeostasis, gluconeogenesis, glycolysis, protein synthesis, amino acid levels, energy metabolism; *vesicles*: membrane trafficking, lysosomes, vesicles; *signaling*: cell signaling (mitogen-activated protein kinase (MAPK), p53, p38 MAPK), signal transduction, and *genotoxicity*: DNA damage, DNA repair, nucleic acid metabolism.

### Epigenomics

DNA methylation and histone acetylation studies do not provide pathway regulation pattern as output and are, therefore, not included in Table 1. They can, however, be used in toxicological screening and data be linked to phenotypic data obtained by classic genotoxicity assays. DNA hypomethylation has been reported after cellular exposure to SiO_2_, ZnO, TiO_2_, CuO, and Ag NPs [35–38] but effects on global DNA methylation in vivo by CuO NPs, Au NPs and SWCNTs are modest [39, 40]. Promoter methylation is increased by 60 nm Au NPs and decreased by SWCNTs in blood cells after intratracheal application of the particles [40]. NPs modify histones by binding to SH groups of histone deacetylases, decreasing the enzymatic activity (Au NPs) [41], or inducing histone hypoacetylation in breast cancer cells (cadmium tellurite (CdTe) quantum dots) [42]. The available data suggest that exposure to NPs may favor cell transformation and tumor development. Conventional phenotypic genotoxicity assays for chromosome damage, for instance COMET assay or micronucleus assay, show variable and partially conflicting results. TiO_2_ particles in sizes < 100 nm showed positive results in COMET assay (17/24), micronucleus (12/16), and sister chromatid exchanges (2/2) but predominantly negative results in COMET (3/5) and micronucleus (2/3) in vivo studies [43]. Also ZnO NPs showed genotoxic action in cellular but not in in vivo studies [44], while studies of SiO_2_ NPs reported weak genotoxic action in vitro and no genotoxicity in animal studies [44]. Lastly, CuO and Ag NPs showed genotoxicity in vitro [45–47] and in vivo [48, 49]. Variation in fibre length, contamination with heavy metals and pre-treatment of the CNTs samples prevent inter-study comparison. It appears that long CNTs induce genotoxicity, while short CNTs do not induce prominent genotoxicity [50]. Phenotypic assays and epigenetic assays identified more damage in cellular than in animal studies. Pathway regulation of DNA damage and repair and nucleic acid metabolism as indication for genotoxicity was also more frequently reported in in vitro than in in vivo studies. The higher frequency of epigenetic changes than of genotoxic effects indicates that epigenomic changes induced by SiO_2_ and TiO_2_ NPs may not result in manifest chromosome damage because repair mechanisms could prevent it. It is, however, possible that the particles act as a challenge making cells more vulnerable to the action of other genotoxic agents.

## Particle effects according to transcriptomics, proteomics and metabolomics studies

Reported regulated pathways were classified according to phenotype changes as routine parameters in toxicity testing. This has the limitation that information on the regulated genes, proteins or metabolites is lost and that high quality and low quality studies are treated equally. Studies using transcriptomics (50) and proteomics (33) were more numerous than studies reporting metabolomics (10) and miRNomics (4) data (Table 1). The number of reports on cellular transcriptomics (37), proteomics (27), metabolomics (7), and miRNomics (4) was higher than that of in vivo studies using these techniques (13 in transcriptomics, 6 in proteomics, and 3 in metabolomics, Table 2). One meta-analysis of gene regulation after pulmonary exposure to CNTs (3) and to TiO_2_ NPs (2) was identified [51]. Transcriptomics studies focused on Ag, SiO_2_, and ZnO NPs, while Au and CNTs were most intensely investigated by proteomics (Tables 1, 2). Pathways were reported with different frequencies in the omics studies. Proliferation, oxidative stress, and immune pathways were mainly affected according to transcriptomics in cells (Table 1). Regulation of inflammation according to transcriptomics was also frequently reported in the in vivo studies of NP exposure by the pulmonary route (Table 2), while more variable regulation pattern were seen after oral and intravenous application. In miRNomics studies various pathway regulations have been reported but due to the low number of miRNomics studies a preference for specific regulation pattern would not be easy to discern. Proteomics of cells reported oxidative stress, morphology, energy metabolism, mitosis and apoptosis as most affected by NP exposure (Table 1). When particles were applied by pulmonary and dermal routes in animals regulation of inflammation was reported (Table 2). Metabolomic studies reported NP effects on oxidative stress, energy metabolism, apoptosis and other pathways in cell exposures (Table 1). In the in vivo studies effects on inflammation were identified (Table 2).

Several studies assessed NPs by omics techniques and phenotypic assays. Usually, only cytotoxicity screening assays was performed to determine the concentration range for the omics studies. This is important because strongly cytotoxic concentrations should be avoided as dead cells provide only limited information on regulatory mechanisms. If, on the other hand, concentrations far outside the toxic range are used, no changes in regulation will be seen. Effects at different particle concentrations were recorded in some studies because reaction to low and high particle concentrations may differ. Comparison with complementary techniques can confirm omics results and support relevance of the reported regulation pattern. Studies combined plate reader analysis of cytokine secretion, mitochodrial activity, cell death, and ROS generation [52–64], flow cytometry for cell cycle analysis and cell death [57, 65–68], microscopy for morphology and immunocytochemical staining [52, 57, 59, 67–73], and genotoxicity assays [74–77] with omics techniques. Other researchers used verification of the regulated pathways by using another omics technique [52, 78, 79]. Influencing the regulated pathway by addition of an antioxidant or studying cell recovery after removal of the particle challenge confirmed involvement of oxidative stress [80–83]. Histology [84–89], analysis of bronchoalveolar lavage fluid [85, 90, 91] and clinical chemistry [84] were performed to support the results of the omics in vivo studies.

The majority of transcriptomic studies evaluated samples up to 24 h, while proteomics studies mainly collected sample at ≥ 24 h (Table 1). mRNA is produced in oscillatory manner and the collection time of the sample is not representative for the levels before and after this time [92]. To avoid this bias, most transcriptomics studies analysed samples at different time points. The majority of proteomics and metabolomics studies, on the other hand, analysed only one time point.

The comparison with another omics technique showed that endoplasmatic reticulum stress induced by Au NPs could be demonstrated on genetic and protein level [78]. Regulated genes and proteins did not overlap in cells exposed to TiO_2_ NPs and MWCNTs [93] but regulated pathways were essentially the same. Similarly, gene regulation coincided very well with exoproteome profiles obtained by exposure of A549 cells with 12 nm SiO_2_ particles [78]. Concordant pathway regulation pattern was also obtained in transcriptomics and proteomics studies of macrophages exposed to Cu, CuO, and TiO_2_ NPs [61, 77]. The sensitivity of the different omics technologies may however differ. Gioria et al. used proteomics and metabolomics and identified different regulation profiles of 5 and 30 nm Au NPs only by proteomics [59].

Inflammation and oxidative stress were reported with different frequencies in transcriptomics and proteomics studies. Different exposure concentrations in the studies is not very likely the explanation as little dose dependency in the regulated genes was observed for SiO_2_ NPs [78]. TiO_2_ and ZnO particles also caused similar regulation pattern over a wide range of concentrations (5–50 µg/ml for TiO_2_ and 0.5–5 µg/ml for ZnO [94]. Another influencing factor may be exposure time. A time-dependent transcriptomics study of various NPs showed that upon short contact with NPs (1–3 h), cells reacted to different types of NPs in a similar way. After 24 h a particle-specific reaction pattern was seen [93]. When only studies are included, where ≥ 3 particles were evaluated (SiO_2_, TiO_2_, CNTs) it is also found that immune effects are reported in the transcriptomics but not in the proteomics in vitro studies. The comparison between transcriptomics and proteomics data from TiO_2_ rods and MWCNTs by Tilton et al. confirmed that regulation of immune processes was more pronounced in gene regulation than in protein regulation. Apparently, NP-induced changes in transcripts of inflammatory genes do not obligatorily result in changes of protein levels [93]. The reduced reporting of immune regulation in proteomics studies is also seen in the in vivo studies on TiO_2_ exposures (Table 2). NPs were applied by different routes and inflammation was reported in 5/7 transcriptomics and 2/5 proteomics studies, while oxidative stress related pathways were identified in 1/7 transcriptomics and 4/5 proteomics studies. The comparison is subjected to certain bias. The 5 proteomics studies were published by only two research groups. Lack of regulation of inflammation in transcriptomics was seen in studies where effects in organs far from the application of the NPs were analysed, in specific, changes in the ovary following intragastral application and changes in the liver of offspring after intratracheal application of the NPs to pregnant mice. The metaanalysis by Nikota et al. on TiO_2_ NPs and CNT confirmed regulation of inflammation but not of oxidative stress by transcriptomics [51]. Data integration is a critical and relevant factor for the outcome of omics studies. Most researchers use Ingenuity Pathway Analysis (IPA) that allows handling of transcriptomics, proteomics and metabolomics data. Free software programs (e.g. Integrated Molecular Pathway Level Analysis, IMPaLA) have similar capacities to analyze data obtained by all these techniques [95]. IMPALA and iPEAP (integrative Pathway Enrichment Analysis Platform) also allow to identify additional pathways from combined datasets originating from different omics techniques. The common software systems integrate data either based on pathway or biochemical ontology, on biological networks or on analysis of empirical correlations [96]. The analysis programs vary in outcome of the analysis (e.g. identification of additional pathways, functional enrichment analysis, differential correlation analysis, etc.), accepted inputs (e.g. genomic, proteomic, metabolomic, biochemical platform independent), user platform (e.g. software, web-based) and difficulty to use. Empirical correlation analysis is usually based on R package and more difficult to perform than pathway enrichment analysis.

The relatively high number of transcriptomics studies on cellular effects of 20 nm Ag NPs can show to which extent study results using the same technology, particles and cells vary. Different pathway regulation pattern were reported by six groups that evaluated the effect of 20 nm Ag NPs by transcriptomics. All of them analysed samples at various time points and all but one included 24 h as a sampling point. Two studies evaluated human dermal fibroblasts at high concentrations and two others studies HepG2 hepatocytes at low concentrations. In the fibroblast studies, but not in the HepG2 studies, there was overlap in the reported pathways. Interestingly, both studies on fibroblasts did not report regulation of oxidative stress, which was reported in most of the other studies on Ag NPs. Effects on fibroblasts were verified by phenotypic assays in one study of fibroblast and in one of the studies on HepG2 cells. Taking into consideration that interarray reproducibility may be low [17], disparate results can be due to the use of different array or pathway analysis platforms. In case of identification of additional pathways, as in the fibroblasts study, sensitivity of the cells could be different. Regarding the HepG2 studies, the different sampling times (≤ 24 h vs. ≥ 24 h) and different exposure concentrations may explain the difference. The comparison may indicate that transcriptomics data are particularly sensitive to the exposure conditions.

As particle handling and biological parameters (passage of cells, preparation of particles, exposure, use of exposure medium) may influence the results, only studies that included more than one particles or > 1 cell type were analysed to reveal particle- or cell-specific regulation pattern.

### Influence of cell types

Cells differ in their resistance to oxidative stress, in the proliferation rate, in the reaction to inflammatory stimuli and their reaction to NPs, e.g. CNTs [97]. Particularly for particles, the ability of cells for phagocytosis appears to be important. Phagocytes ingest NPs to a higher degree and the particle accumulation may affect the physiology of phagocytes more than that of epithelial cells [98]. The reported regulation patterns, however, do not support this hypothesis as similar patterns were published for macrophages and epithelial cells exposed to ZnO NPs [94, 99]. Phagocytosis also appears not to be regulated by exposure to NPs. Only two proteomics and one transcriptomics studies reported regulation of phagocytosis [93, 100, 101], while the majority of transcriptomics (6) and proteomics (5) studies did not report this. The low importance of the cell type in pathway regulation was corroborated by a meta-analysis on regulation in Caco-2, THP-1, and small airway cells by TiO_2_ and CNTs. The authors concluded that regulation pattern were more particle-specific than cell-specific [93].

Differences in contact between particles and cells growing either adherent or non-adherent, may affect regulation. Although cells growing in suspension culture also settle on the bottom of the plate after a certain time, they do not form confluent monolayers and this may lead to different exposure doses. Differences in cellular particle uptake between adherent and non-adherent growing cells have been reported for instance for CNTs [102]. The reduced uptake could explain the different cytotoxicity of CNTs in cells growing in suspension compared to adherent cells [97]. Consistent with the hypothesis of a different reaction of adherent and non-adherent cells, alumina NPs showed a dose-dependent and time-dependent increase in cytotoxicity for adherent cells but only dose-dependent increase for suspension cells [103]. The majority of cells that were used in the studies (Table 1) were adherent growing cells and only two studies analysed cells growing in suspension in parallel to adherent growing cells [94, 104]. Fe_3_O_4_ were tested in THP-1 monocytes (growing in suspension) and in adherent growing HepG2 hepatocytes by transcriptomics. Since lack of contact may be a reason for a decreased cellular response, cellular uptake of the particles was determined by Prussian Blue staining. Despite lower uptake by THP-1 cells, more genes than in HepG2 cells were regulated, which suggests a higher sensitivity of the immune cells to exposure to Fe_3_O_4_ particles. The other transcriptomics study, however, did not identify prominent differences between Jurkat lymphocytes (suspension) and macrophages (adherent growth) when exposed to ZnO NPs. In this case different cell contact due to particle sedimentation was irrelevant because the authors concluded that the actions were caused by dissolved Zn^2+^ ions and not by intact particles.

### Influence of particle properties

Biological effects are influenced by a variety of parameters, mainly by material, size, surface properties, and shape [105]. A particle-specific regulation pattern would not be unexpected but responses to plain particles of different material in transcriptomics and proteomics studies (e.g. Fe_2_O_3_/SiO_2_/ZnO; WC/WCCo; SWCNT/MWCNT; TiO_2_/CuO; ZnO/ZrO) in a given cell line after ≥ 24 h were uniform [53, 77, 100, 101, 106, 107]. Although the proteomics study on the effect of Au, Cu and CdTe NPs in THP-1 cells suggested particle-specific regulation [108], other studies do not give indication for particle-specific regulation in RAW 264.7 macrophages [61, 77, 101]. After pulmonary application, C_60_ fullerenes and NiO NPs regulated particle-specific and common transcriptomic pathways in mouse lungs [55]. Furthermore, all omics studies of pulmonary application of NPs, irrespective of the material, reported regulation of immune system and inflammation. These results support the hypothesis of a cell-/organ-specific reaction pattern. It might be possible that the invasive application, in general intratracheal instillation, increased the propensity for inflammation. The absence of pulmonary inflammation after inhaled TiO_2_ NPs versus instilled NPs supports this assumption [109].

When particles in different sizes and surface properties were studied the following can be concluded. Transcriptomics studies identified mainly quantitative differences in the regulation by particles of different size. Typically, smaller particles caused an effect, while the larger particles did not (e.g. [80], Table 1). Different pathways (oxidative stress vs. cell signaling), by contrast, have been reported for 20 and 100 nm Au NPs in proteomics [60]. Surface qualities did not markedly influence the regulation pattern according to transcriptomics and proteomics studies. Cellular effects of differently functionalized Au particles and bare and differently functionalized Fe_3_O_4_ particles in transcriptomics as well as action of coated and plain TiO_2_ and plain and pegylated SWCNTs support the missing effect of surface properties (Table 1). Comparative metabolomics study on intratracheally instilled polystyrene and polymer particles demonstrated a correlation between surface hydrophobicity and extent of the inflammatory reaction. This finding is consistent with results obtained by conventional testing where particles with hydrophobic surface induced higher immune response than those with hydrophilic surface [110].

Studies of spherical and rod-like CuO suggest a small influence of shape on gene regulation, with rod-like NPs showing a stronger pro-inflammatory effect than spherical particles [111]. Also the meta-analysis by Tilton et al. concluded that exposure to TiO_2_ rods and CNTs induced a particle-specific regulation pattern [93]. This leads to the hypothesis that particle-specific regulation may occur for non-spherical compared to spherical particles.

In summary, particle parameters caused rather quantitative than qualitative differences in the regulation pattern.

## Correlation to phenotypic assays

For evaluation of the use of omics technologies in toxicity testing of NPs it is important to know the extent to which pathway regulation corresponds to phenotypic changes. The best method for this comparison is the choice of a phenotypic assay platform capable to analyse multiple parameters in the same cell population. Conventional screening comprises a panel of colorimetric, fluorometric and luminescent test methods for the detection of apoptosis, membrane damage, proliferation, lysosome function, etc. in parallel exposures. Interference of NPs may occur in one or more of these assays [112, 113]. High-content screening systems (HCS) have the advantage that they combine various fluorescent assays with detection of morphological changes by bright field microscopy. This way, several parameters can be analysed in parallel and inconsistency between signal and cell morphology can be discerned.

The suggested assay panel representing the most common targets for a comprehensive analysis of NP toxicity included: (i) cytotoxicity (proliferation, membrane leakage and integrity, ATP content, mitochondrial potential, metabolic activity, calcium flux, apoptosis), (ii) genotoxicity by DNA cleavage (micronucleus assay), (iii) inflammation (interleukin 1, 8 or tumor-necrosis factor alpha, nuclear factor kappa B, or activator protein-1 activation), (iv) oxidative stress (ROS generation or GSH), and (v) fibrotic potential (tumor growth factor-1 beta, collagens 1 and 3 and metalloproteinase activity) [114]. These categories correspond in essence to the pathway regulation classes in Table 1. Differences include the lack of fibrotic potential and genotoxic potential in the table and the addition of proliferation, morphology, vesicles and signaling pathways. Another set of targets for the toxicity screening of NPs, namely proliferation, apoptosis, inflammation and genotoxicity, has also been suggested [115].

The available HCS data are ambiguous regarding cell-specific reaction to NPs. CdTe NPs induced quantitatively different responses in differentiated and undifferentiated murine neuronal cells. Human and murine neuroblastoma cells, neural progenitor cells and neural stem cells reacted in different way to iron oxide NPs. Furthermore, 50 nm amine-functionalized polystyrene NPs induced apoptosis in a variety of cells (astrocytes, HEK293, A549, HepG2, and hMECD) but not in RAW 264.7 macrophages. Lack of cell-specific action, on the other hand was reported by other studies. Membrane damage and mitochondrial damage induced by TiO_2_, CeO_2_, and ZnO in sizes between 5 and 20 nm was similar in BEAS-2B and macrophages [116–119] and 35 nm Fe_3_O_4_ NPs produced the same profile in murine fibroblasts and simian COS cells [120].

The potential of screening by phenotypic assays is limited in the identification of new modes of action. Except for the cytotoxicity screening assays, they can only detect a specific cellular effect and the characterization of particle effects depends on the selection of the right assays. This can be seen as disadvantage compared to omics techniques in the untargeted form.

## Conclusions

Omics platforms could be useful to identify new pathways and mechanisms in nanotoxicity not visible in conventional testing. This is, however, not always the case for NPs. Studies of polystyrene particles identified corresponding targets by conventional assays and whole genome transcription arrays [53, 105, 121]. Transcriptomic analysis, on the other hand, identified adverse cellular effects at lower concentrations than conventional cytotoxicity screening based on ATP content, dehydrogenase activity and cell impedance monitoring [78]. The comparison is complicated by the fact that the regulation of genes indicates a potential damage but does not prove that cell damage will actually occur. Researchers reported different regulation patterns by similar NPs in the same cells tested with the same technologies. As omics data were confirmed by phenotypic assays, disparate results between research groups may be caused by different exposure conditions. More frequently reported regulation of inflammation in cellular transcriptomics than proteomics studies, on the other hand, may be linked to the technology. General (technology-independent) problems with in vitro testing of NPs and issues related to omics technologies that limit their application in nanotoxicity testing are listed in Table 3. Different particle exposure conditions have been suspected to be the reason for inter-study differences in phenotypic assays. In order to avoid this problem, standard operation procedures (SOPs) for preparation of particle suspensions, use of cell lines and preparation of cells have been developed (see for instance overview https://www.nanopartikel.info/nanoinfo/methodik/401-arbeitsanweisung). The general use of these SOPs by all researchers may decrease variations between studies. The use of confirmatory assays (e.g. another omics technique, phenotypic assays) is important to demonstrate study quality and verify pathway regulation.

**Table 3:** Limitations that hinder the broad use of omics technologies in nanotoxicology

Independent from the technology	Technology linked
Lack of standardization of particle exposure	Request for high sample quality (freezing, protection against degradation)
Sample pre-treatment	Expertise in bioinformatics needed for data analysis
Cell type used for testing	Lack of standardization of sample preparation
Medium composition	Predictive value of the omics techniques not entirely clear
Relevant concentration range	

## Abbreviations

Ag: silver
Au: gold
BAL: bronchoalveolar lavage fluid
CdTe: cadmium tellurite
CNT: carbon nanotube
Co: cobalt
Cu: copper
DCs: dendritic cells
Diff.: differentiated
ECM: extracellular matrix
ER: endoplasmic reticulum
ESI: electrospray ionization
Exp.: exposure
Fe_x_O_y_: iron oxide
FGF-2: fibroblast growth factor 1
FTIR: Fourrier transform ion cyclotron resonance
GC: gas chromatography
GSH: glutathione
HCS: high content screening
HGF: hepatic growth factor
HPLC: high performance liquid chromatography
HSP: heat shock protein
id: intradermal
ig: intragastral
IMPaLA: Integrated Molecular Pathway Level Analysis
inhal: inhalation
ip: intraperitoneal
IPA: Ingenuity Pathway Analysis
iPEAP: integrative Pathway Enrichment Analysis Platform
it: intratracheal instillation
iv: intravenous
LC: liquid chromatography
MALDI: matrix-assisted laser desorption ionization
MAPK: mitogen activated protein kinase
MSCs: mesenchymal stem cells
MS: mass spectrometry
MWCNT: multi-walled carbon nanotube
NfkB: nuclear factor kappa B
NMR: nuclear magnetic resonance
NP: nanoparticle
oroph: oropharyngeal aspiration
ox.: oxidative
PAK: p21 activated kinases
RISC: RNA-induced silencing complex
SDS: sodium dodecyl sulfate
SiO_2_: silicium oxide
SOP: standard operation procedures
SWCNT: single-walled carbon nanotube
TFG-β: tumor growth factor beta
Tech: technique
TiO_2_: titanium dioxide
TOF: time of flight
WC: tungsten carbide
ZnO: zinc oxide
ZrO: zirconium oxide
